# The Least Understood Placental Galectins: Galectin-14 and Galectin-16 in Human Pregnancy and Placental Biology

**DOI:** 10.3390/diagnostics16193188

**Published:** 2026-09-30

**Authors:** Zoltan Kozinszky, Yakov Rachamin Karo, Jakó Mária, Márió Vincze, János Sikovanyecz, János Sikovanyecz, Zsolt Tidrenczel, Bálint Koppány, Giuseppe Gullo, László Kaiser, Eszter Ducza, Szabolcs Várbíró, Andrea Surányi

**Affiliations:** 1 Department of Obstetrics and Gynecology, Albert Szent-Györgyi Clinical Center, University of Szeged, H-1134 Szeged, Hungaryvincze.mario92@gmail.com (M.V.);; 2 Capio Specialized Center for Gynecology, 171 45 Solna, Sweden; 3 Department of Obstetrics and Gynecology, Genetic Center, Hungarian Defence Forces Medical Center, H-1134 Budapest, Hungarytidrenc@hotmail.com (Z.T.);; 4 Department of Obstetrics and Gynecology, Villa Sofia-Cervello Hospital, University of Palermo, 90100 Palermo, Italy; gullogiuseppe@libero.it; 5 Department of Pathology, University of Szeged, H-6725 Szeged, Hungary; 6 Department of Pharmacodynamics and Biopharmacy, Faculty of Pharmacy, University of Szeged, H-6720 Szeged, Hungary; ducza.eszter@szte.hu

**Keywords:** galectin-14, galectin-16, LGALS14, LGALS16, placenta, trophoblast, maternal–fetal interface, pregnancy complications

## Abstract

Galectins represent a family of proteins which are capable of specifically binding to beta-galactoside-containing sugar molecules, known as glycans. Galectins possess carbohydrate recognition domains (CRDs) that fundamentally determine their ability to bind to sugars. Galectin-14 (Gal-14) and galectin-16 (Gal-16) are members of the galectin family that is particularly associated with the placenta. The galectins in the placenta constitute a specialized subgroup functioning at the maternal–fetal interface. They are expressed primarily in the syncytiotrophoblasts. This trophoblast layer is in direct contact with maternal blood and plays a crucial role in the metabolic, hormonal and immunological interactions between the fetus and the pregnant woman. In order to prepare this structured narrative review, we conducted a structured literature search across the MEDLINE (PubMed), Embase, Cochrane Library and Web of Science Core Collection databases; the final search has been performed on 1 July 2026. The search strategy has combined the terms galectin-14/LGALS14 and galectin-16/LGALS16 with terms related to the placenta, trophoblast, pregnancy, placentation and pregnancy complications. Search results were imported into Mendeley Reference Manager to remove duplicates, and the reference lists of relevant review articles and studies were manually screened. Two reviewers (Z.K. and A.S.) independently screened the titles and abstracts and subsequently evaluated the potentially relevant full-text articles. The final evidence synthesis included primary studies that directly investigated the expression, function, regulation, or clinical associations of LGALS14 or LGALS16; secondary publications were used for background and contextual information. In experimental studies, galectin-14 has been linked to trophoblast migration and invasion—involving mechanisms such as the PI3K/Akt signaling pathway, MMP-9 and N-cadherin—as well as to the modulation of activated maternal immune cell function. Galectin-16 is structurally more distinct and exhibits reduced classical β-galactoside-binding capacity; it appears to be closely associated with trophoblast differentiation and syncytialization, presumably through intracellular protein interactions. Altered expression of placenta-specific galectins has been observed in preeclampsia and fetal growth restriction; however, the causal relationship remains unclear. Clinical evidence regarding their use as biomarkers is emerging at this time; therefore, neither galectin-14 nor galectin-16 can be currently considered as a validated, clinically applicable biomarker. Altogether, available evidence mandates further study of galectin-14 and galectin-16, as they may be important regulators of placental biology and potential biomarker candidates. However, their physiological functions and clinical utility have not yet been sufficiently confirmed; therefore, further prospective and functional studies are required. The strongest putative mechanistic link to the development of preeclampsia exists for galectin-14, primarily involving trophoblast migration and invasion as well as PI3K/Akt-related signaling pathways. In contrast, evidence in connection with the direct role of galectin-16 is limited at present.

## 1. Introduction

Galectins, as an evolutionarily conserved family of soluble β-galactoside-binding proteins, translate glycan information into cellular responses through the conserved carbohydrate recognition domain (CRD). Fifteen human galectins are classified into three distinct types based on how their CRDs are organized: (1) prototype galectins contain one single CRD and often form homodimers (e.g., Galectin-1, -7, -10); (2) tandem-repeat galectins contain two distinct, linked CRDs on a single peptide chain (e.g., Galectin-4, -8, -9); (3) chimera-type galectins contain a single CRD that is attached to a unique non-lectin N-terminal tail (represented solely by Galectin-3) [1,2,3,4].

Their functions comprise the regulation of cell adhesion, migration, apoptosis, immune responses, angiogenesis and tissue remodeling [2,5,6]. Most galectins lack a classical N-terminal signal peptide and can therefore function both in extracellular and intracellular spaces. Extracellular galectins can cross-link glycosylated receptors and extracellular matrix components, whereas intracellular galectins may act through protein–protein interactions independent of carbohydrate recognition [2,4,7]. These properties are particularly relevant in the placenta, but the present review focuses on the specialized biology of LGALS14 and LGALS16 rather than the galectin family in a broad sense [8,9,10,11,12,13,14,15,16,17,18,19,20,21,22].

At the maternal–fetal interface, galectin-14 and galectin-16 are notable for their highly restricted trophoblast expression [3,23,24,25,26]. Placental galectins can participate in trophoblast differentiation, syncytialization, invasion, interactions with the extracellular matrix, as well as immune regulation [3,23,25]. Among galectins located on chromosome 19 (Chr19), LGALS14 and LGALS16 are particularly enriched in differentiated trophoblasts, especially syncytiotrophoblasts. Their localization at the maternal–fetal interface makes them plausible regulators of trophoblast function and communication with maternal immune and vascular compartments. However, the functions of LGALS14 and LGALS16 should not simply be inferred from better-characterized family members [3,23,24,27,28,29,30,31,32,33,34,35,36].

LGALS14 and LGALS16 form a distinctive Chr19 cluster, similar to LGALS13. Comparative genomic evidence supports lineage-specific gene duplication and diversification within this cluster in anthropoid primates [3,23,37,38,39]. Their restricted phylogenetic distribution and trophoblast expression suggest functional specialization associated with primate placentation. Galectin-14 has been associated with trophoblast migration, invasion and immune cell regulation, whereas galectin-16 appears more closely related to trophoblast differentiation and has unusual carbohydrate-binding properties [3,23,37]. Major unanswered questions concern their molecular targets, regulation, intracellular versus extracellular functions and significance in pregnancy disorders. Therefore, this review focuses on what is known at present and clearly distinguishes direct evidence from proposed mechanisms [39,40]. Findings derived directly from experimental or clinical studies must be distinguished from interpretations based on expression patterns, structural characteristics, pathway associations, or analogy with other galectins.

## 2. Methods

A structured literature search supporting this narrative review was carried out on 1 July 2026 in MEDLINE via PubMed, Embase, the Cochrane Library and the Web of Science Core Collection. No filters were applied based on language or study design. Publication status was indicated as ‘accepted for publication’, ‘in press’, or ‘published’. The search strategy combined two principal concepts and used controlled vocabulary, including Medical Subject Headings (MeSH), where applicable, together with free-text terms. The search topic consisted of the following terms: ‘galectin-14’ OR ‘galectin 14’ OR ‘LGALS14’ OR ‘galectin-16’ OR ‘galectin 16’ OR ‘LGALS16’, combined with placental and pregnancy terms including ‘placenta’ OR ‘placental’ OR ‘trophoblast’ OR ‘pregnancy’ OR ‘gestation’ OR ‘placentation’ OR ‘maternal-fetal interface’. A complementary complication-focused search combined the terms for galectin with ‘preeclampsia’ OR ‘pre-eclampsia’ OR ‘fetal growth restriction’ OR ‘FGR’ OR ‘intrauterine growth restriction’ OR ‘IUGR’ OR ‘recurrent pregnancy loss’ OR ‘miscarriage’ OR ‘pregnancy loss’ OR ‘gestational diabetes’ OR ‘gestational diabetes mellitus’ OR ‘preterm birth’ OR ‘preterm delivery’ OR ‘placental insufficiency’. Database-specific syntax was adapted to the respective controlled vocabulary and field structure. The review was conducted in accordance with the Scale for the Assessment of Narrative Review Articles (SANRA) recommendations [41].

Records retrieved from the databases were imported into Mendeley Reference Manager for data deduplication. Reference lists of relevant reviews and the included primary studies were also screened manually to ensure thorough coverage and to identify potentially relevant articles not retrieved by the electronic searches.

### Study Selection

Mechanistic observations originating from different experimental systems were interpreted in the context of their source models, whereas observational associations and proposed mechanisms were distinguished from direct experimental evidence. Accordingly, uniform evidentiary weight was not attributed to the available evidence, and causal interpretations were avoided where the available data did not support them.

**Figure 1:**
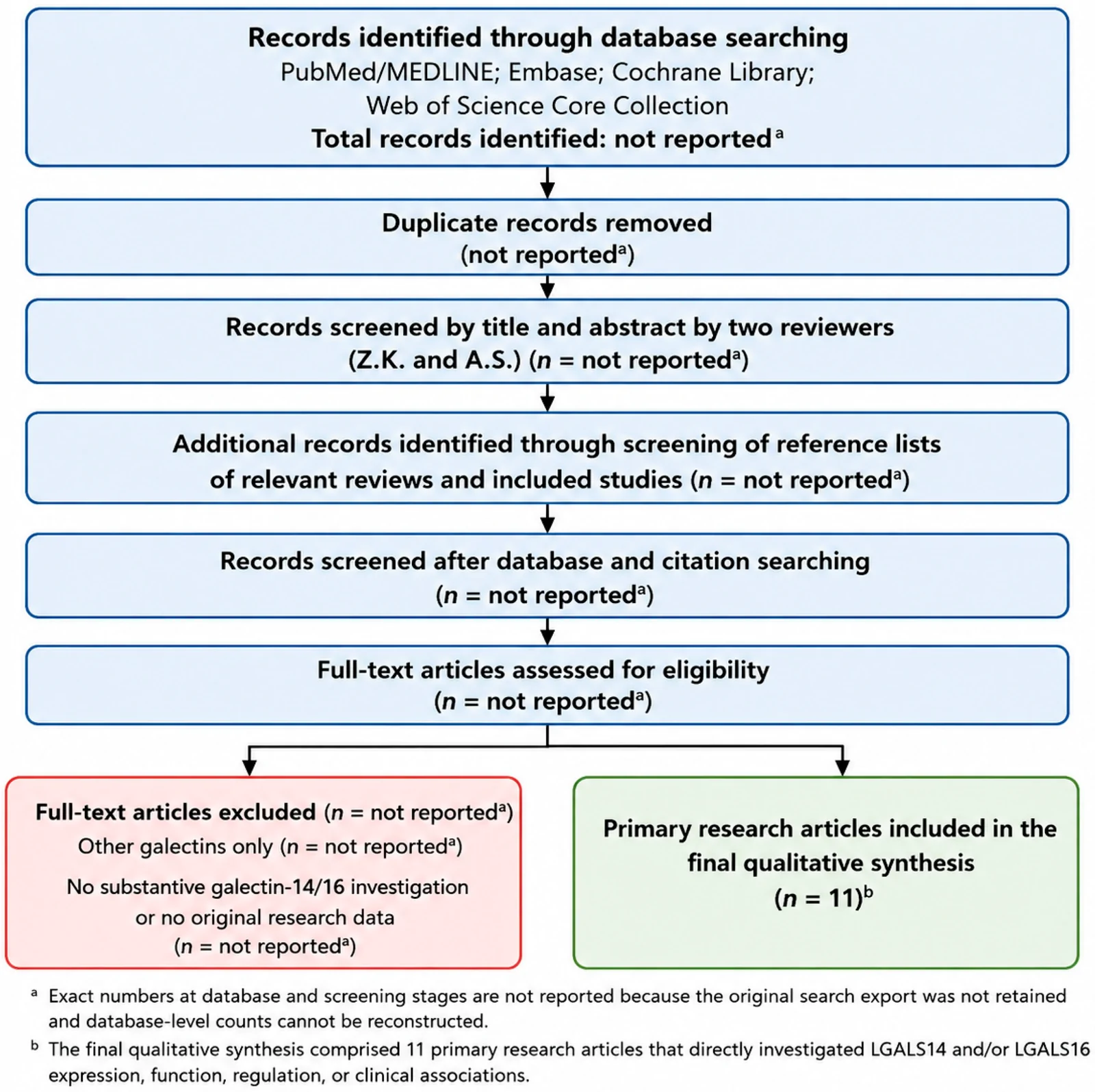
Structured literature identification and study selection.

Two reviewers (Z.K. and A.S.) screened the retrieved records independently in two stages: title/abstract screening followed by full-text assessment of potentially eligible articles. Disagreements were resolved by discussion and consensus. Primary research articles were eligible when they provided original experimental or clinical evidence concerning galectin-14 or galectin-16 in human pregnancy, placental biology, trophoblast function, or pregnancy-related disorders. Reviews, editorials, methodological papers and other secondary publications were retained for contextual background but were not taken into consideration as primary studies during the final evidence set. Full-text articles were excluded when galectin-14 or galectin-16 was not substantively investigated, when the article was unrelated to placental biology or pregnancy, or when no original research data were presented. The final qualitative synthesis was composed of 11 primary research articles, in which LGALS14 and/or LGALS16 expression, function, regulation, or clinical associations were directly investigated. The literature identification and study selection process is summarized in Figure 1.

## 3. Chr19 Cluster Galectin Genes: Evolutionary Context

LGALS14 and LGALS16 are members of the galectin cluster at 19q13.2 on chromosome 19. The evolutionary history described below serves as background information based on comparative genomic studies.

### 3.1. Evolutionary Background of Chr19 Cluster Galectin Genes

Galectins (LGALS13, LGALS14 and LGALS16) on Chromosome 19 form a distinct subgroup with expression restricted almost exclusively to the placenta through primate-specific gene duplication and diversification.

Their restricted phylogenetic distribution suggests that their emergence occurred in parallel with reproductive characteristics of anthropoid primates, including deep hemochorial placentation, prolonged gestation and complex maternal–fetal immune interactions [3,23,38,42].

The galectin cluster on chromosome 19 is consistent with the birth-and-death evolution model of multigene families. According to this model, gene duplication generates new copies that subsequently undergo sequence divergence, functional specialization, or pseudogenization. The duplicated genes may acquire novel functions while retaining structural characteristics inherited from their ancestral gene. In the case of Chr19 galectins, this process appears to have been accompanied by both coding-sequence diversification and the acquisition of regulatory elements that promoted placenta-specific expression [16,23,38,39].

Evolutionary diversification appears to have involved regulatory mechanisms in addition to changes in protein sequence. Comparative genomic and transcriptomic analyses reveal that Chr19 galectins possess regulatory elements associated with trophoblast-specific expression. Recent single-cell and transcriptomic studies further demonstrate that LGALS13, LGALS14 and LGALS16 are predominantly expressed in syncytiotrophoblasts and other differentiated trophoblast populations, whereas expression in most adult non-placental tissues is minimal or absent. This highly restricted pattern suggests that changes in cis-regulatory elements were important for establishing their specialized placental functions [3,24,25,43,44,45].

### 3.2. Molecular Evolution of Galectin-14 and Galectin-16

Galectin-14 and galectin-16 originated from relatively recent duplication events within the chromosome 19 galectin cluster and subsequently underwent distinct molecular evolution. Although both proteins retain the characteristic galectin fold and a single carbohydrate recognition domain, comparative structural analyses indicate that sequence divergence has affected their carbohydrate-binding properties and potentially their biological functions [3,23,37].

Galectin-14 appears to have retained many of the structural characteristics associated with conventional prototype galectins. In contrast, galectin-16 has accumulated amino acid substitutions within and around the canonical carbohydrate-binding pocket. Structural studies indicate that these changes substantially reduce its affinity for β-galactosides compared with classical galectins. This divergence raises the possibility that galectin-16 has undergone neofunctionalization toward biological mechanisms that are less dependent on conventional extracellular glycan recognition [37,46].

Available evidence indicates that galectin-14 and galectin-16 evolved through coordinated changes in protein structure and transcriptional regulation. Galectin-14 appears to have retained more conventional galectin characteristics, whereas galectin-16 shows greater divergence in its carbohydrate-binding domain and may have shifted toward intracellular or glycan-independent functions [37,46]. This evolutionary divergence provides an important molecular basis for investigating their distinct roles in human placental biology [3,7,37]. These differences create a framework for the generation of functional hypothesis: sequence and structural divergence may hint at functional specialization, while differences in regulatory elements and trophoblast-specific expression may indicate stage- or cell-state-dependent roles. However, evolutionary divergence alone does not create biological function and should be regarded as a basis for testable hypotheses rather than evidence of established physiological activity.

## 4. Molecular Characteristics and Expression of Galectin-14 and Galectin-16

**Figure 2:**
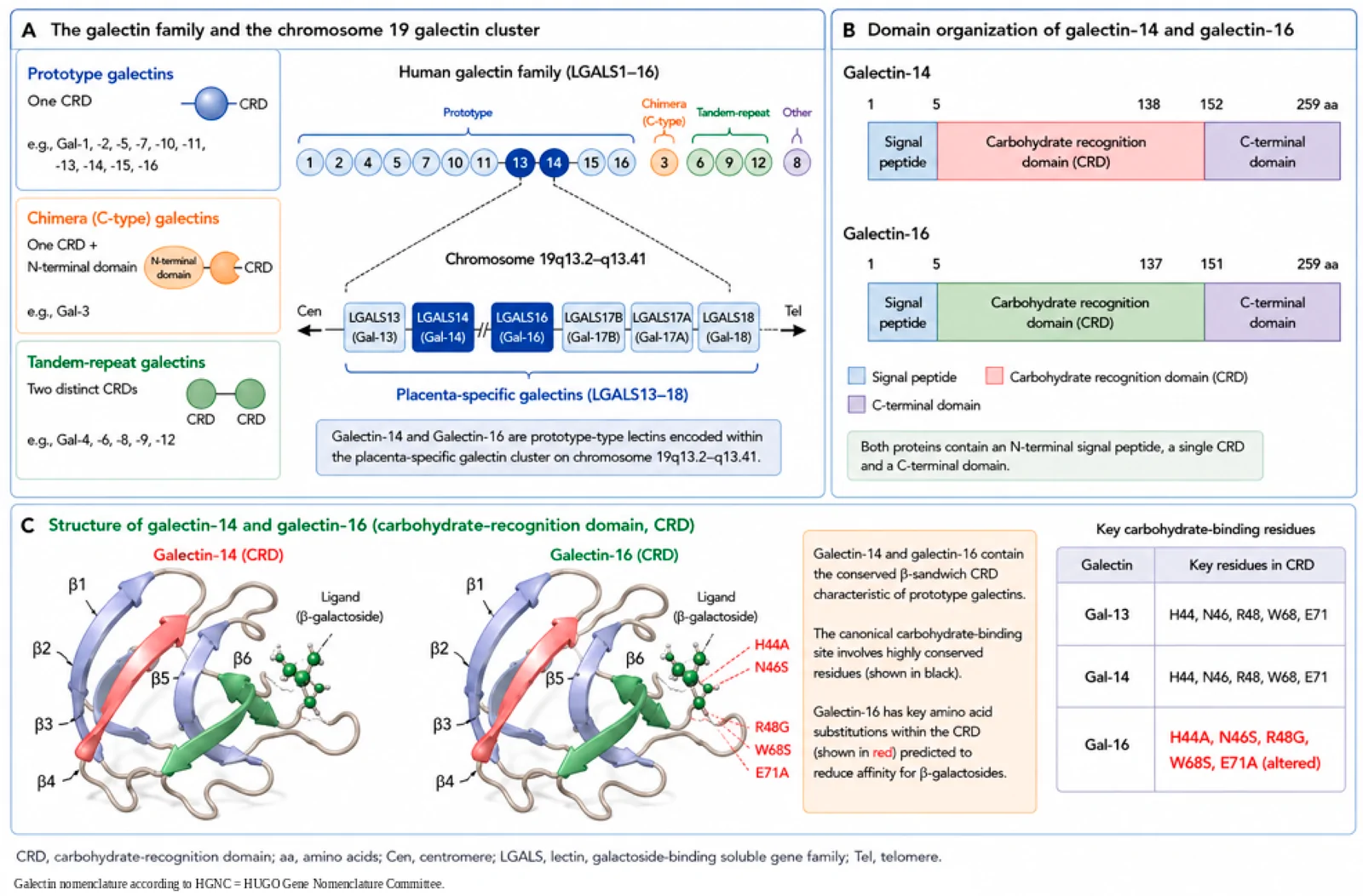
Evolution and molecular characteristics of placenta-specific galectins, Galectin-14 and Galectin-16.

The close genomic organization of galectin-14 (LGALS14) and galectin-16 (LGALS16) reflects the tandem duplication events that contributed to the evolution of this placenta-specific galectin subgroup (Figure 2). Despite their shared evolutionary origin, LGALS14 and LGALS16 have acquired distinct coding and regulatory characteristics that may contribute to differences in their molecular functions [3,23]. The principal comparative characteristics of galectin-14 and galectin-16 as well as selected human galectins are summarized in Table 1.

Both proteins belong to the prototype galectin subgroup and contain a single carbohydrate recognition domain (CRD) consisting of approximately 130 amino acids [2,22,47]. Galectin-16 exhibits unusual structural differences compared to other members of the galectin family, including galectin-14. While galectin-14 conforms to the canonical galectin model, galectin-16 is distinguished by its unique structural and functional characteristics: recombinant galectin-16 exists primarily as a monomer, and its ability to form oligomers under physiological conditions remains uncertain [37]. These unique structural features suggest that galectin-16 functions primarily through glycan-independent pathways [2,4,7]. Both galectin-14 and galectin-16 lack an N-terminal signal peptide, indicating that they are likely secreted via non-conventional pathways, thereby allowing them to exert their effects both inside and outside the trophoblast cells [2,4,7]. Whether these structural differences result in entirely distinct physiological roles for the two proteins is a key area for future research.

### Regulation of Galectin-14 and Galectin-16 Expression

Research findings regarding transcriptional and developmental regulation have been published in several experimental studies. These studies address the underlying regulatory mechanisms directly. The expression of LGALS14 and LGALS16 is strictly confined to placental trophoblasts and is closely linked to trophoblast differentiation. Their expression is governed by specific regulatory pathways that emerge during placental development [3,23,24].

Recent experimental results offer more direct evidence regarding the regulation of LGALS16 during trophoblast differentiation. During cAMP-induced syncytialization, LGALS16 expression increases in parallel with markers of syncytiotrophoblast maturation. Pharmacological studies suggest that this response is primarily mediated by the cAMP-directly activated exchange protein (EPAC) and p38 mitogen-activated protein kinase (MAPK) signaling pathways, rather than by classical protein kinase A (PKA) signaling. These observations imply that multiple cAMP-dependent signaling contributes to the transcriptional regulation of LGALS16 during trophoblast differentiation [48].

On the whole, recent data indicate that the expression of LGALS14 and LGALS16 is determined by a combination of trophoblast-specific transcriptional regulation, developmental signaling and potentially epigenetic and environmental mechanisms.

## 5. Placental Expression Patterns of Galectin-14 and Galectin-16

**Figure 3:**
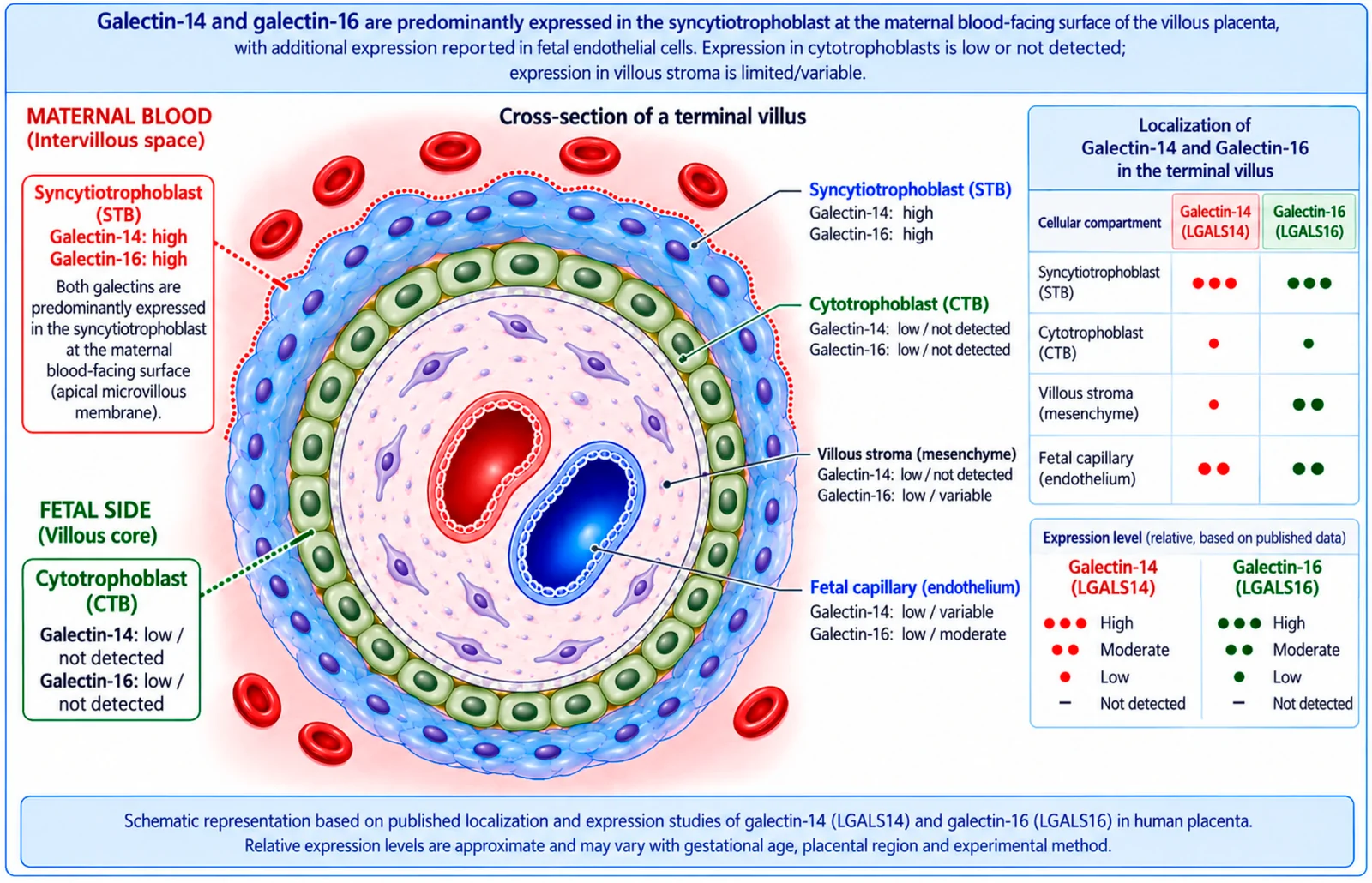
Placental localization of Galectin-14 and Galectin-16.

Galectin-14 and galectin-16 exhibit highly restricted expression within the human placenta, distinguishing them from more broadly expressed galectins such as galectin-1 and galectin-3. Their expression is predominantly associated with trophoblast populations, particularly syncytiotrophoblasts, which represent the principal cellular interface between maternal blood and fetal placental tissue [3,23,24] (Figure 3).

The increasing use of single-cell RNA sequencing and spatial transcriptomics has provided more detailed information about the cellular distribution of placenta-specific galectins. These studies demonstrate that LGALS13, LGALS14 and LGALS16 are strongly enriched within trophoblast populations, particularly in differentiated trophoblasts, while displaying minimal or absent expression in most maternal immune cells, stromal cells and fetal endothelial cells. This highly restricted transcriptional pattern supports their classification as characteristic molecular components of human trophoblasts and provides further evidence for specialized functions at the maternal–fetal interface [3,25,26].

The cellular localization of galectin-14 and galectin-16 also offers important clues to their potential biological functions. Their enrichment in syncytiotrophoblasts may point to their possible roles in maintaining placental barrier function, cellular homeostasis and in communication with maternal blood. Expression in cytotrophoblasts and potentially in extravillous trophoblasts (EVT) additionally raises the possibility of functions related to trophoblast differentiation, migration and tissue remodeling. Nevertheless, localization alone cannot establish biological function; thus, functional studies are required to determine whether these proteins actively regulate the affected processes.

### Temporal Expression Pattern During Pregnancy

The expression of placenta-specific galectins changes during gestation in connection with placental development and trophoblast differentiation. The placenta undergoes continuous structural and functional remodeling throughout pregnancy, with trophoblast proliferation, differentiation and syncytialization accompanied by substantial changes in gene expression. The temporal regulation of LGALS14 and LGALS16 therefore implies that their functions may vary according to the developmental stage of the placenta [3,23].

Available evidence suggests that LGALS14 and LGALS16 expression is particularly associated with trophoblast maturation and syncytiotrophoblast differentiation. The increase in LGALS16 during cAMP-induced syncytialization provides direct experimental support for this relationship and indicates that its expression is integrated into the molecular pathways governing trophoblast fusion [48].

Another unresolved question is whether galectin-14 and galectin-16 are released from trophoblast cells into the maternal circulation. Galectins can be secreted through unconventional pathways, and placenta-derived extracellular vesicles provide an additional mechanism through which trophoblast proteins may enter maternal blood circulation. If galectin-14 or galectin-16 are released in biologically significant quantities, circulating concentrations can potentially deliver information about placental function. At present, however, evidence corroborating their use as circulating biomarkers remains insufficient [3,23].

Available evidence cumulatively indicates that galectin-14 and galectin-16 exhibit highly restricted trophoblast expression and are closely associated with placental differentiation and maturation. Their predominant localization in syncytiotrophoblasts, together with the evidence of developmental regulation, supports a role in specialized placental functions. However, the precise temporal trajectories of both proteins throughout normal pregnancy and their alterations in pregnancy complications remain insufficiently defined. Longitudinal studies combining tissue, single-cell and circulating measurements will be required to explore their physiological expression patterns and determine whether deviations from these patterns have clinical significance.

## 6. Biological Functions of Galectin-14 and Galectin-16

Galectin-14 and galectin-16 are increasingly recognized as multifunctional, placenta-specific proteins that may play role in trophoblast differentiation, migration and invasion, maternal–fetal immune regulation, placental vascular development and the maintenance of tissue homeostasis. It is appropriate to examine their biological functions within the context of validated experimental findings and emerging mechanistic hypotheses [3,7,23,37].

For galectin-14, experimental observations strongly attest to extracellular or cell-associated effects linked to trophoblast migration and invasion—involving the PI3K/Akt signaling pathway, MMP-9 and N-cadherin—as well as effects on activated maternal immune cells. In contrast, galectin-16 exhibits significantly reduced default β-galactoside-binding activity and is more closely associated with trophoblast differentiation and syncytialization; evidence for potential intracellular, glycan-independent functions remains to be generated.

Table 2 summarizes the primary evidence in respect of galectin-14 and galectin-16, elucidating key biological and clinical associations. In this chapter, we draw a distinction between experimentally verified effects and putative or proposed functions. Observations derived from specific cellular, biochemical, structural, or clinical models were not qualified as evidence of physiological or causal effects, which extend beyond the directly examined systems.

### 6.1. Immunomodulatory Functions at the Maternal–Fetal Interface

Galectin-14 has been shown to interact with activated peripheral blood mononuclear cells (PBMCs) and, particularly, activated T lymphocytes. Experimental exposure to galectin-14 can induce apoptosis in activated immune cells in a concentration-dependent manner. This feature suggests that galectin-14 may contribute to limiting excessive maternal immune activation at the maternal–fetal interface, potentially supporting local immune tolerance [19,23,53].

Galectin-14 also affects cytokine production. Experimental studies have reported changes in cytokines, including IL-8, IL-10 and IFN-γ following exposure to galectin-14. These findings indicate that its immunological effects cannot simply be characterized as generalized immunosuppression. Galectin-14 may modulate the composition and intensity of the local cytokine environment, with potentially different effects, depending on the activation state of responding immune cells [3,23]. These observations were obtained ex vivo or in cell-based experimental systems and therefore demonstrate effects under test conditions without ascertaining that galectin-14 would exert the same effects at the maternal–fetal interface in vivo. The biological significance of these findings in human pregnancy remains uncertain and requires validation in physiologically relevant placental and maternal–fetal models.

Knowledge about the immunomodulatory effects of galectin-16 is much more limited. Its strong trophoblast-specific expression and evolutionary relationship with galectin-14 provide a rationale for investigating its potential role at the maternal–fetal interface, but direct evidence for effects on maternal immune cells remains limited. It is currently unknown whether galectin-16 has overlapping immunological functions with galectin-14 or has developed a distinct mechanism related to its divergent molecular structure.

### 6.2. Regulation of Angiogenesis and Placental Vascular Development

Galectin-14 may influence angiogenesis indirectly through its effects on trophoblast function. In addition, galectin-14-induced changes in IL-8 production are of interest, because IL-8 can promote endothelial migration and angiogenic responses [23,54].

Direct evidence for galectin-16 in placental angiogenesis is even more limited. Its predominant localization within differentiated trophoblasts nevertheless places it in a cellular compartment capable of regulating the angiogenic microenvironment.

### 6.3. Regulation of Apoptosis, Cell Survival and Tissue Homeostasis

Galectin-14 appears to have effects on cellular survival. In trophoblast cells, activation of PI3K/Akt may promote cell survival, proliferation and migration, whereas in activated maternal T lymphocytes, galectin-14 can induce apoptosis. This apparent duality may reflect the different receptor profiles and intracellular signaling environments of trophoblasts and immune cells [3,23,55].

Less is known about galectin-16 in cell survival and apoptosis. Its association with syncytiotrophoblast differentiation suggests that it may contribute to the maintenance of mature trophoblast function, but direct evidence remains insufficient. Since syncytiotrophoblasts form a long-lived multinucleated cell layer exposed continuously to maternal blood, mechanisms maintaining its integrity and responding to cellular stress are particularly important. Whether galectin-16 contributes to these processes remains an open question.

### 6.4. Cell Signaling and Intercellular Communication

In the case of galectin-14, activation of the PI3K/Akt pathway links the protein to trophoblast migration, invasion and survival, as well as extracellular matrix remodeling; thus, it may represent a central signaling mechanism through which galectin-14 influences placental development [23].

Galectin-16 may represent a more unusual signaling model. Its reduced standard β-galactoside-binding activity suggests that extracellular glycan recognition may not fully explain its biological functions. Recent structural and biochemical evidence emphasizes the possibility that galectin-16 can interact with intracellular proteins and may therefore regulate cellular processes through protein–protein interactions rather than by classical galectin-mediated glycan lattices [7,20,21,37].

An interaction between galectin-16 and the NF-κB family member c-Rel has raised the possibility that galectin-16 could influence transcriptional programs involved in immune regulation and trophoblast differentiation. However, the downstream consequences of this interaction remain incompletely defined, and independent validation is required before it can be considered an established physiological pathway.

## 7. Galectin-14 and Galectin-16 in Pregnancy Complications

The putative role of galectin-14 and galectin-16 in pregnancy complications is most evident in the context of preeclampsia; for this condition, available experimental and clinical data can be integrated into a cautiously formulated mechanistic framework. Altered expression of the * LGALS14 * gene has been reported in preeclampsia. The significance of galectin-16 may originate from its role in trophoblast differentiation and syncytialization, although evidence regarding its direct contribution to preeclampsia remains limited.

The placenta-specific expression and the biological activity of galectin-14 and galectin-16 call for investigation of their roles in pregnancy complications characterized by abnormal placentation, impaired trophoblast differentiation, altered maternal–fetal immune regulation and placental dysfunction. These conditions include, among others, preeclampsia, fetal growth restriction (FGR), recurrent pregnancy loss (RPL), gestational diabetes mellitus (GDM) and preterm birth [3,23,24,25,28,56]. The evidence with regard to these specific conditions, such as trophoblast biology, preeclampsia and pregnancy loss is not uniform.

### 7.1. Preeclampsia

Preeclampsia is a heterogeneous pregnancy disorder characterized by abnormal placentation, impaired spiral artery remodeling, placental stress, systemic inflammation and maternal endothelial dysfunction. In the early-onset form of the disease, defective trophoblast invasion and inadequate transformation of the uterine spiral arteries are particularly important components of placental pathology. Given the close association of galectins with trophoblast differentiation and function, altered expression of these proteins may indicate abnormalities in placental development [57,58].

Among placenta-specific galectins, galectin-14 has attracted particular interest. Experimental studies indicate that galectin-14 can promote trophoblast migration and invasion through activation of the PI3K/Akt pathway and increased expression of MMP-9 and N-cadherin. Reduced galectin-14 activity could therefore theoretically contribute to impaired trophoblast invasion and extracellular matrix remodeling, processes that play a central role in the pathogenesis of early-onset preeclampsia [3,23].

Clinical and placental studies have also reported altered expression of galectin-14 in pregnancies with preeclampsia. Reduced LGALS14 gene expression has been described particularly in severe or preterm preeclampsia, and differential methylation of LGALS14 has been recorded in case of villous trophoblasts from the affected pregnancies. These findings raise the possibility that altered transcriptional regulation of Chr19 galectins contributes to the abnormal trophoblast phenotype observed in preeclampsia [3,23,50].

A clear distinction must hence be made between association and causality. Reduced expression of galectin-14 or galectin-16 may be the result of impaired trophoblast differentiation, altered oxygenation, inflammatory signaling, or epigenetic changes that occur during preeclampsia. Conversely, reduced galectin activity itself can contribute to impaired trophoblast function and thereby exacerbate placental dysfunction. Prospective studies that begin in the first trimester and are combined with functional experimental models are needed to distinguish between these possibilities.

### 7.2. Fetal Growth Restriction

Fetal growth restriction is a clinically heterogeneous condition in which the fetus fails to achieve its growth potential. Placental insufficiency, abnormal trophoblast invasion, impaired uteroplacental vascular development and maternal vascular malperfusion are important factors in many cases of FGR [52,59].

The proposed functions of galectin-14 provide several possible connections to FGR. Galectin-14 promotes trophoblast migration and invasion and regulates MMP-9 and N-cadherin through PI3K/Akt signaling. Impairment in these processes could contribute to inadequate trophoblast-mediated remodeling of the maternal circulation and consequently to reduced placental perfusion. However, direct evidence connecting galectin-14 signaling to the development of FGR remains limited [3,23].

Altered expression of Chr19 galectins has been reported in placentas affected by growth restriction, although findings are not sufficiently consistent to declare that galectin-14 or galectin-16 are independent markers of FGR. This limitation is important because placental gene expression can be influenced by gestational age, fetal sex, placental location, maternal disease and the severity and time course of placental insufficiency.

Galectin-16 could theoretically be relevant in the pathogenesis of FGR through its association with syncytiotrophoblast differentiation and placental homeostasis. Future studies should investigate galectin-14 and galectin-16 in well-phenotyped FGR cohorts, distinguishing early-onset disease from late-onset disease as well as placental causes from non-placental causes. Integration of placental transcriptomics, protein localization, maternal circulating biomarkers, and placental histopathology could help us to determine whether these galectins would provide valuable information in addition to the established markers of placental dysfunction.

### 7.3. Recurrent Pregnancy Loss

Galectin-14 is of particular interest because it can influence activated maternal immune cells. Experimental studies have demonstrated that galectin-14 induced apoptosis of activated T lymphocytes and changes in cytokine production, suggesting its possible role in regulating maternal immune responses at the maternal–fetal interface [23]. Galectin-14 may additionally contribute to implantation through its effects on trophoblast migration and extracellular matrix remodeling. Inadequate galectin-14 expression or activity could therefore theoretically affect both sides of the implantation process: trophoblast competence and maternal immune adaptation. Reduced galectin-14 expression has been described in some pregnancy-loss settings, supporting the necessity for further investigation [3,23,51].

The evidence for galectin-16 in recurrent pregnancy loss is considerably weaker. Because LGALS16 expression is strongly associated with trophoblast differentiation, altered expression could potentially reflect abnormal early placental development. However, there is currently insufficient evidence to determine whether galectin-16 contributes directly to pregnancy loss or whether altered expression is secondary to defective trophoblast differentiation.

### 7.4. Gestational Diabetes Mellitus

Placental adaptations to maternal hyperglycemia can affect trophoblast function, nutrient transport, oxidative stress and fetal growth [60].

Although galectins have been implicated generally in metabolic regulation and inflammation, direct evidence concerning galectin-14 and galectin-16 in GDM remains limited. Their highly restricted trophoblast expression and involvement in placental homeostasis provide a biological rationale for investigating whether their expression changes in pregnancies complicated by maternal metabolic dysfunction.

Future clinical studies should combine maternal metabolic phenotyping with placental galectin expression and neonatal outcomes. Such an approach could determine whether galectin-14 or galectin-16 are merely altered in response to GDM or whether specific placental phenotypes associated with fetal overgrowth, growth restriction, or other adverse outcomes could be identified.

### 7.5. Preterm Birth

Preterm birth can be characterized by multiple etiological mechanisms. Given that galectins participate in immune regulation and trophoblast biology, several members of the galectin family have been investigated in relation to spontaneous as well as medically indicated preterm birth.

Galectin-14 may be crucial due to its effects on maternal immune cell apoptosis and cytokine production. Changes in these processes could potentially influence the inflammatory environment at the maternal–fetal interface. Its effects on trophoblast migration and tissue remodeling can forge an additional link to placental integrity [23].

Galectin-16 could potentially be involved through its association with syncytiotrophoblast differentiation and cellular homeostasis. However, direct evidence connecting either galectin-14 or galectin-16 to spontaneous preterm birth remains insufficient.

This distinction is particularly important because preterm birth represents several biologically different phenotypes. The galectin profile associated with infection-driven preterm birth may differ from the galectin profile associated with placental insufficiency or medically indicated preterm delivery associated with preeclampsia.

### 7.6. Other Pregnancy-Related Disorders

Altered expression of placenta-specific galectins has also been investigated in other disorders involving abnormal placentation, inflammation and maternal–fetal communication. These include placental inflammatory conditions, placenta accreta spectrum and other forms of placental dysfunction. However, evidence specifically implicating galectin-14 or galectin-16 remains sparse and is predominantly observational.

The most convincing mechanistic data pertain to galectin-14, as well as trophoblast migration, invasion and immune cell regulation. Evidence for galectin-16 remains substantially provisional and is currently focused on trophoblast differentiation and its unusual molecular properties (Table 2).

A major priority for future research is therefore to distinguish altered galectin expression as a biomarker of placental disease from a genuine pathogenic mechanism. Prospective longitudinal cohorts, first-trimester sampling, spatial and single-cell molecular profiling, placental organoids and functional gene-editing approaches will be essential for determining whether galectin-14 and galectin-16 contribute to disease development or simply reflect the altered cellular state of the placenta under pathological conditions.

At present, the evidence supporting galectin-14 and galectin-16 as clinical biomarkers is limited to exploratory and observational studies, without sufficient prospective validation of diagnostic accuracy, prognostic performance, or incremental clinical value.

## 8. Clinical Applications and Biomarker Potential

The highly restricted expression of galectin-14 and galectin-16 in placental trophoblast makes them attractive candidates for the development of biomarkers reflecting placental differentiation and function. However, the clinical evidence supporting their use as biomarkers remains limited, and neither galectin-14 nor galectin-16 can currently be considered as established clinical biomarker [3,23,24,61].

### 8.1. Potential as Biomarker of Placental Function

A major challenge in obstetric medicine is the early identification of pregnancies in which placental dysfunction will subsequently result in complications such as preeclampsia, fetal growth restriction, or fetal loss. Current clinical markers often identify disease only after substantial placental dysfunction has already developed. Placenta-derived proteins that are detectable in maternal blood could potentially provide earlier information about trophoblast differentiation, placental stress or abnormal placentation.

Galectin-14 and galectin-16 are particularly interesting in this context because of their highly restricted placental expression. If changes in placental expression are reflected in maternal circulation, their concentrations could potentially serve as minimally invasive indicators of placental activity. Galectin-14 has already attracted interest in relation to placental dysfunction and pregnancy complications, whereas evidence concerning circulating galectin-16 is substantially less compelling [3,23].

The interpretation of circulating galectin concentrations nevertheless requires caution. A change in maternal serum concentration may reflect altered transcription, protein synthesis, secretion, release of extracellular vesicles, protein stability, or maternal clearance. Consequently, circulating measurements cannot automatically be regarded as a direct substitute for expression in placental tissue. Longitudinal studies comparing maternal blood concentrations with placental transcript and protein expression are required to describe these relationships.

The timing of sampling may also be of critical importance. Placental galectin expression changes with trophoblast differentiation and gestational age, meaning that the concentration considered abnormal in one trimester may be physiological in another. First-trimester studies may be particularly informative because molecular alterations preceding clinical manifestations of preeclampsia or FGR could potentially be detected before manifest placental dysfunction develops.

### 8.2. Integration with Multi-Omics and Liquid Biopsy Approaches

Recent advances in placental transcriptomics, single-cell RNA sequencing, spatial transcriptomics and proteomics have substantially improved the ability to characterize cell-specific molecular changes in pregnancy. Integration of LGALS14 and LGALS16 expression with these datasets could help us to determine which trophoblast populations express these proteins and how their expression changes during normal and pathological placental development [3,24,25,43,44,45,62,63].

Maternal liquid biopsy may provide an additional opportunity. Placental extracellular vesicles, cell-free RNA and other placenta-derived molecular signals can be detected in maternal blood and may provide complementary information about placental function. If galectin-14 or galectin-16 are released in association with extracellular vesicles or other placenta-derived particles, their detection could potentially be incorporated into multimarker approaches rather than be used as isolated biomarkers [64,65].

A combined biomarker panel may ultimately be more informative than a single measurement of galectin. Galectin-14 or galectin-16 could theoretically be integrated with established angiogenic markers, inflammatory mediators, placental proteins and clinical characteristics to improve prediction of specific obstetric syndromes. This approach would also allow the biological heterogeneity of disorders such as preeclampsia and FGR to be taken into account.

### 8.3. Potential Therapeutic Relevance

The biological effects of galectin-14 and galectin-16 also raise the possibility that these proteins or their signaling pathways could become therapeutic targets. Principally, the modulation of galectin activity could influence trophoblast invasion, differentiation, immune regulation, or placental homeostasis. However, therapeutic targeting remains highly speculative because the physiological functions of these proteins are not yet sufficiently understood.

Galectin-14 is particularly interesting because experimental evidence links it to PI3K/Akt signaling, trophoblast migration and invasion and maternal immune cell regulation. Manipulating this pathway could theoretically influence placental development, but indiscriminate enhancement or inhibition could also have undesirable effects because trophoblast invasion must remain strictly under control. Excessive trophoblast invasion is associated with a different spectrum of placental disorders than inadequate invasion.

Galectin-16 presents an even greater therapeutic challenge. Its unusual carbohydrate-binding properties and possible intracellular functions have the consequence that extracellular inhibition or supplementation may not adequately modify its biological activity. Identification of its intracellular binding targets and downstream pathways will therefore be necessary before rational therapeutic strategies can be concluded [7,37].

**Figure 4:**
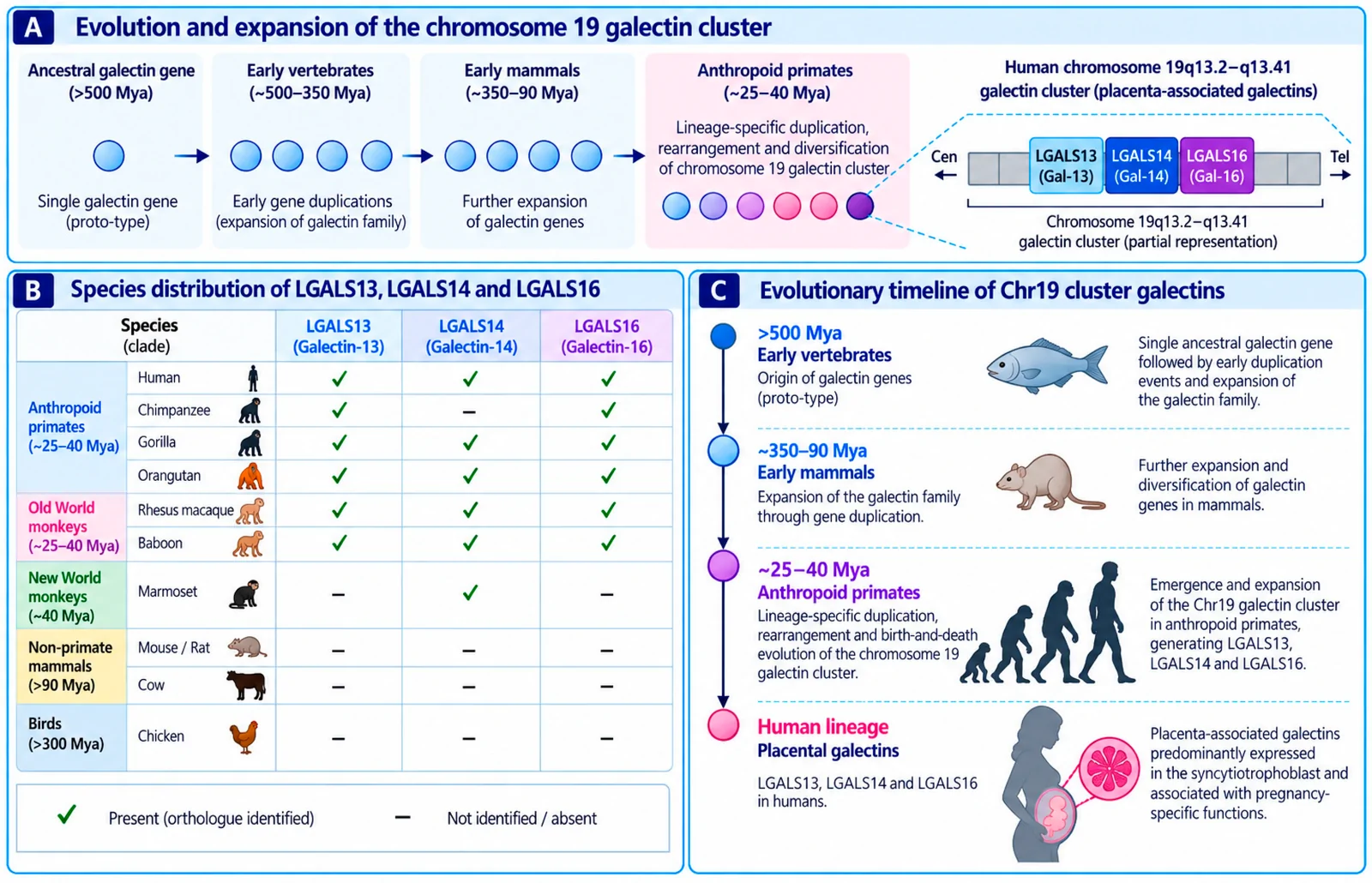
Evolutionary context of Chr19 cluster galectins. Schematic representation of the evolutionary diversification and genomic organization of the chromosome 19 galectin cluster, including *LGALS13*, *LGALS14*, and *LGALS16*, with comparative species distribution and an evolutionary timeline [38,40,42].

The lineage-specific evolutionary history of Chr19 galectins represents an additional translational limitation (Figure 4). Conventional rodent models cannot fully reproduce the biology of human galectin-14 and galectin-16. Human trophoblast cell systems, placental organoids, ex vivo placental tissue and humanized experimental models may therefore be particularly important for preclinical validation.

All in all, galectin-14 and galectin-16 have considerable potential as molecular indicators of placental biology, but their clinical application is still premature.

## 9. Knowledge Gaps and Future Perspectives

Despite increasing interest in placenta-specific galectins, substantial gaps remain in our understanding of galectin-14 and galectin-16. Compared with better-characterized members of the galectin family, particularly galectin-1 and galectin-13, relatively few studies have directly investigated their molecular interactions, cellular functions and physiological significance. Consequently, several proposed functions of galectin-14 and galectin-16 remain preliminary and require independent experimental validation [3,7,23,37].

One of the most important unresolved questions concerns the molecular partners of these proteins. For galectin-14, the mechanisms linking extracellular binding to PI3K/Akt activation, MMP-9 expression and trophoblast migration remain incompletely defined. The receptors or glycan structures responsible for its effects on maternal immune cells are also unknown. Identifying these molecules would help determine whether the observed effects represent direct galectin-mediated signaling or secondary consequences of altered cellular interactions [3,23,66].

For galectin-16, the central question is fundamentally different. Structural studies indicate reduced canonical β-galactoside-binding activity, raising the possibility that its biological functions depend predominantly on intracellular protein–protein interactions. The reported interaction with c-Rel provides an intriguing starting point, but the physiological relevance of this interaction and its downstream consequences remain unclear. Comprehensive identification of galectin-16 binding partners is therefore a major priority [7,37,66].

The regulation of LGALS14 and LGALS16 expression also requires further investigation. Although trophoblast-specific transcriptional programs, GCM1, TFAP2 family members, epigenetic mechanisms and developmental signaling pathways have been implicated, direct functional evidence remains limited. It is also unclear how oxygen tension, inflammatory cytokines, metabolic signals and endocrine factors modify expression during normal pregnancy and in placental disease. Defining these regulatory mechanisms could explain why galectin expression changes in pathological pregnancies [3,23].

Another important gap concerns the precise cellular and spatial distribution of galectin-14 and galectin-16 throughout gestation. Single-cell and spatial transcriptomic studies have established strong trophoblast enrichment, but transcript abundance does not necessarily predict protein localization or biological activity. Longitudinal studies combining single-cell transcriptomics, spatial transcriptomics, proteomics and high-resolution immunolocalization could determine how expression changes during trophoblast differentiation and across different placental regions.

The extracellular biology of these proteins is similarly poorly understood. Galectins can be secreted through unconventional pathways and may interact with extracellular glycoconjugates, cell-surface receptors or extracellular matrix components. It remains uncertain whether galectin-14 and galectin-16 are released into maternal blood in biologically meaningful concentrations and, if so, whether circulating protein originates predominantly from the placenta. Extracellular vesicles represent a further potential mechanism of transport and communication between trophoblast and maternal tissues, but direct evidence for functional transport of galectin-14 or galectin-16 through these vesicles remains limited [3,23].

The relationship between altered galectin expression and pregnancy complications also requires clarification. Reduced or increased expression in preeclampsia, FGR, or other disorders may represent a cause of placental dysfunction, a compensatory response, or simply a consequence of altered trophoblast differentiation. Cross-sectional studies cannot adequately distinguish these possibilities. Prospective studies beginning in early pregnancy, with serial maternal sampling and subsequent placental phenotyping, are therefore particularly important.

A further challenge is the evolutionary specificity of chromosome 19 galectins. Because LGALS14 and LGALS16 are characteristic of anthropoid primates, conventional rodent models cannot fully reproduce their physiological biology (Figure 4). Human trophoblast cell models, primary trophoblast cultures, placental explants, trophoblast organoids and placenta-on-chip systems may therefore provide more appropriate experimental platforms. Genome editing, including CRISPR/Cas-based approaches, could allow direct assessment of the consequences of LGALS14 or LGALS16 loss or overexpression.

Modern multi-omics approaches provide an opportunity to integrate these experimental systems. Combining transcriptomics, spatial profiling, proteomics, glycoproteomics and interactomics could define the molecular networks associated with galectin-14 and galectin-16 and distinguish direct molecular targets from downstream effects. Integration with detailed placental histopathology may additionally establish whether particular galectin signatures correspond to specific pathological patterns of maternal or fetal vascular malperfusion, inflammation or abnormal trophoblast development.

Finally, the potential clinical relevance of these proteins requires prospective validation. Before galectin-14 or galectin-16 can be considered clinically useful biomarkers, trimester-specific reference ranges, analytical reproducibility and diagnostic performance must be established in adequately powered cohorts. Their incremental value over established clinical, biochemical and ultrasound markers should also be determined. Similarly, therapeutic targeting should not be pursued until their physiological functions and tissue-specific effects are sufficiently understood.

Overall, future research should move from descriptive expression studies toward mechanistic and longitudinal investigation. The most important priorities are identification of molecular interactors, characterization of regulatory pathways, validation in human placental models, determination of circulating biology and prospective assessment in clinically well-defined pregnancy cohorts. These approaches may establish whether galectin-14 and galectin-16 represent functional regulators of human placentation, biomarkers of placental dysfunction, or both.

Despite increasing interest in placenta-specific galectins, substantial gaps remain in our understanding of galectin-14 and galectin-16. Compared with better-characterized family members like galectin-1 and galectin-13, relatively few studies have directly investigated their molecular interactions, cellular functions and physiological significance [3,7,23,37]. Consequently, several proposed functions for both proteins remain undiscovered and require independent experimental validation. Key areas requiring further investigation include molecular targets of galectin-14 [3,23]. The mechanisms linking extracellular binding to PI3K/Akt activation, MMP-9 expression and trophoblast migration remain incompletely defined. Additionally, the specific receptors or glycan structures responsible for its effects on maternal immune cells are unknown [7,37]. Identifying these molecules is critical to determining whether these effects represent direct galectin-mediated signaling or just the secondary consequences of altered cellular interactions. Structural studies indicate reduced default β-galactoside-binding activity in intracellular interaction of galectin-16, suggesting that its biological functions may depend predominantly on intracellular protein–protein interactions. While the reported interaction with c-Rel provides an intriguing starting point, its physiological relevance and downstream consequences remain unclear. Comprehensive identification of binding targets of galectin-16 is a major priority. Regulation of LGALS14 and LGALS16 expression requires further study. Although trophoblast-specific transcriptional programs (including GCM1 and TFAP2 family members), epigenetic mechanisms and developmental signaling pathways have been implicated, direct functional evidence remains limited. It is also unclear how microenvironmental factors, such as oxygen tension, inflammatory cytokines, metabolic signals and endocrine factors, modulate their expression.

## 10. Conclusions

Galectin-14 and galectin-16 are placenta-specific proteins that are encoded on chromosome 19. These galectins play distinct roles: galectin-14 influences trophoblast migration and immunity, whereas galectin-16 promotes differentiation. However, their physiological significance *in vivo* remains unconfirmed. Their altered expression is associated with preeclampsia and miscarriage, but data regarding fetal growth restriction, gestational diabetes and preterm birth are preliminary or insufficient. Current findings indicate an association rather than a causal relationship, meaning that laboratory data cannot be directly extrapolated to clinical pathology. None of these proteins is an established biomarker or therapeutic target. Future research requires validated human models and prospective clinical trials to confirm their diagnostic value relative to existing markers; until then, their role still remains unproven.

## Figures and Tables

**Table 1:** Comparative characteristics of selected human galectins.

Characteristic	Galectin-13	Galectin-14	Galectin-16	Galectin-1	Galectin-3	Galectin-9	Galectin-10 *
**Gene (LGALS)**	LGALS13	LGALS14	LGALS16	LGALS1	LGALS3	LGALS9	LGALS10
**Chromosomal location**	19q13.2	19q13.2	19q13.2	22q13	14q22	17q11	19q13
**Placenta-specific**	Yes	Yes	Yes	No	No	No	No
**Evolutionary origin**	Anthropoid primates	Lineage-specific diversification in anthropoid primates	Lineage-specific diversification in anthropoid primates	Conserved vertebrate	Conserved vertebrate	Conserved vertebrate	Primate-specific
**Protein type**	Prototype	Prototype	Prototype	Prototype	Chimera	Tandem-repeat	Prototype
**Carbohydrate-binding activity**	Weak/modified	Reduced/modified	Greatly reduced or absent	Strong	Strong	Strong	Lost
**Major placental expression**	Syncytiotrophoblast	Syncytiotrophoblast, cytotrophoblast	Trophoblast	Trophoblast, decidua, endothelium	Trophoblast, macrophages	Trophoblast, decidua, immune cells	Minimal
**Main biological functions**	Immune tolerance, vascular remodeling	Immune regulation, trophoblast differentiation, apoptosis	Trophoblast biology, immune regulation (putative)	Immune tolerance, angiogenesis, invasion	Cell adhesion, inflammation, apoptosis	Immune checkpoint, T-cell regulation	Eosinophil driven inflammation, Charcot–Leyden crystal formation
**Associated pregnancy disorders**	Preeclampsia, FGR, PAS	Preeclampsia, FGR, RPL, GDM (limited evidence)	Preeclampsia, FGR, RPL, GDM (limited evidence)	Preeclampsia, miscarriage, implantation failure	Preeclampsia, GDM, inflammation	Preeclampsia, miscarriage, infection	None confirmed
**Maternal serum biomarker**	Yes	Investigational	Investigational	Yes	Yes	Investigational	No
**Animal models available**	No	No	No	Yes	Yes	Yes	Yes
**Crystal structure available**	Yes	Yes	Predicted/limited structural data	Yes	Yes	Yes	Yes
**Known ligands/receptors**	Annexin II, glycans (reported)	Poorly characterized	Unknown	Integrins, laminin, fibronectin, glycans	Integrins, mucins, TLR4, K-Ras	TIM-3, PDI, CD44, glycans	Not well defined
**Therapeutic potential**			in exploratory phase	High	High	High	Limited

* Galectin-10 is included as a comparative reference; its role is not placenta-specific.

**Table 2:** Summary of primary studies investigating galectin-14 and galectin-16 in human pregnancy and placental disease.

Galectin	Biological or Clinical Background	Number of Primary Studies	Evidence Summary	References
**Galectin-14**	Placental expression/trophoblast biology	8	Eight primary studies directly investigated the placental expression, trophoblast differentiation, migration/invasion, immune regulation or molecular characterization of galectin-14.	[46,49]
**Galectin-14**	Immune regulation	3	Three primary studies directly examined galectin-14 in maternal immune cell or lymphocyte regulation.	[23]
**Galectin-14**	Preeclampsia	3	Three primary studies reported altered galectin-14 expression or mechanistic involvement in preeclampsia.	[50]
**Galectin-14**	Early pregnancy loss/miscarriage	3	Three primary studies reported reduced galectin-14 expression or associations with early pregnancy loss/miscarriage.	[51]
**Galectin-14**	Fetal growth restriction/IUGR/FGR	1	One primary study directly included IUGR/FGR/pathological pregnancies and reported altered LGALS14 expression.	[52]
**Galectin-14**	Gestational diabetes mellitus	0	No primary study directly investigating galectin-14 in GDM could be identified.	—
**Galectin-14**	Preterm birth	0	No primary study directly investigating galectin-14 as a preterm birth biomarker or mechanism could be identified.	—
**Galectin-16**	Placental expression/trophoblast differentiation	5	Five primary studies directly investigated LGALS16 expression, trophoblast differentiation, molecular regulation or structure/function.	[48]
**Galectin-16**	Preeclampsia	1	One primary study directly investigated LGALS16 expression/methylation in preeclampsia; change in expression was not statistically significant, although differential methylation was detected.	[3,50]
**Galectin-16**	Early pregnancy loss/miscarriage	0	No primary study directly investigating galectin-16 in miscarriage/early pregnancy loss could be identified.	—
**Galectin-16**	Fetal growth restriction/IUGR	0	No primary study directly investigating galectin-16 in FGR/IUGR could be identified.	—
**Galectin-16**	Gestational diabetes mellitus	0	No primary study directly investigating galectin-16 in GDM could be identified.	—
**Galectin-16**	Preterm birth	0	No primary study directly investigating galectin-16 in preterm birth could be identified.	—

References are cited in square brackets according to the reference list; “—“ indicates that no directly corresponding reference was identified for that category.

## Data Availability

No new data were created or analyzed in this study. Data sharing is therefore not applicable to this article.

## Abbreviations

aa, amino acids; Akt, protein kinase B; CGB, chorionic gonadotropin beta; Cen, centromere; CRD, carbohydrate recognition domain; EPAC, exchange protein directly activated by cAMP; EVT, extravillous trophoblast; FGR, fetal growth restriction; GDM, gestational diabetes mellitus; HGNC, HUGO Gene Nomenclature Committee; IFN, interferon; IL, interleukin; IUGR, intrauterine growth restriction; LGALS, lectin, galactoside-binding soluble gene family; MAPK, mitogen-activated protein kinase; MeSH, Medical Subject Headings; MMP, matrix metalloproteinase; NF-κB, nuclear factor kappa B; PAS, placenta accreta spectrum; PBMC, peripheral blood mononuclear cell; PI3K, phosphatidylinositol 3-kinase; PKA, protein kinase A; RPL, recurrent pregnancy loss; SANRA, Scale for the Assessment of Narrative Review Articles; Tel, telomere. LGALS14 and LGALS16 are the official human gene symbols; galectin-14 and galectin-16 refer to the corresponding proteins.
